# Negative Immune Checkpoint Protein, VISTA, Regulates the CD4^+^ T_reg_ Population During Sepsis Progression to Promote Acute Sepsis Recovery and Survival

**DOI:** 10.3389/fimmu.2022.861670

**Published:** 2022-03-24

**Authors:** Chyna C. Gray, Bethany Biron-Girard, Michelle E. Wakeley, Chun-Shiang Chung, Yaping Chen, Yael Quiles-Ramirez, Jessica D. Tolbert, Alfred Ayala

**Affiliations:** ^1^ Department of Molecular Biology, Cell Biology, and Biochemistry, Brown University, Providence, RI, United States; ^2^ Division of Surgical Research, Department of Surgery, Brown University, Providence, RI, United States

**Keywords:** Vista, sepsis, regulatory T cells, cytokines, liver injury, Foxp3, CTLA4, CD25

## Abstract

Sepsis is a systemic immune response to infection that is responsible for ~35% of in-hospital deaths and over 24 billion dollars in annual treatment costs. Strategic targeting of non-redundant negative immune checkpoint protein pathways can cater therapeutics to the individual septic patient and improve prognosis. B7-CD28 superfamily member V-domain Immunoglobulin Suppressor of T cell Activation (VISTA) is an ideal candidate for strategic targeting in sepsis. We hypothesized that immune checkpoint regulator, VISTA, controls T-regulatory cells (T_reg_), in response to septic challenge, thus playing a protective role/reducing septic morbidity/mortality. Further, we investigated if changes in morbidity/mortality are due to a T_reg_-mediated effect during the acute response to septic challenge. To test this, we used the cecal ligation and puncture model as a proxy for polymicrobial sepsis and assessed the phenotype of CD4^+^ T_regs_ in VISTA-gene deficient (VISTA^-/-^) and wild-type mice. We also measured changes in survival, soluble indices of tissue injury, and circulating cytokines in the VISTA^-/-^ and wild-type mice. We found that in wild-type mice, CD4^+^ T_regs_ exhibit a significant upregulation of VISTA which correlates with higher T_reg_ abundance in the spleen and small intestine following septic insult. However, VISTA^-/-^ mice have reduced T_reg_ abundance in these compartments met with a higher expression of Foxp3, CTLA4, and CD25 compared to wild-type mice. VISTA^-/-^ mice also have a significant survival deficit, higher levels of soluble indicators of liver injury (i.e., ALT, AST, bilirubin), and increased circulating proinflammatory cytokines (i.e., IL-6, IL-10, TNFα, IL-17F, IL-23, and MCP-1) following septic challenge. To elucidate the role of T_regs_ in VISTA^-/-^ sepsis mortality, we adoptively transferred VISTA-expressing T_regs_ into VISTA^-/-^ mice. This adoptive transfer rescued VISTA^-/-^ survival to wild-type levels. Taken together, we propose a protective T_reg_-mediated role for VISTA by which inflammation-induced tissue injury is suppressed and improves survival in early-stage murine sepsis. Thus, enhancing VISTA expression or adoptively transferring VISTA^+^ T_regs_ in early-stage sepsis may provide a novel therapeutic approach to ameliorate inflammation-induced death.

## 1 Introduction

Despite exhaustive research on sepsis over the last 50 years (1) there remains no effective patho-physiological treatment options nor molecular methods of diagnosis. The incidence of sepsis has not improved, with sepsis accounting for ~35% of non-cardiac deaths during intensive care unit hospitalization, accounting for ~1 in 5 deaths worldwide (2), and it was the consensus cause of death assigned to those dying from COVID-19 infection (3). At >24 billion dollars in annual treatment costs, sepsis presents an economic as well as a healthcare burden (4). Historically, sepsis clinical trials have targeted the initial pro-inflammatory response by inhibiting cytokines in septic patients (5, 6). Efficacy was not universal, and treatment predisposed patients to fatal secondary infections (7).

Immune checkpoint blockade (ICB) has been used to ameliorate disease pathology with greater precision and success than many immune-directed therapies (8). Our laboratory, among others, has demonstrated that negative checkpoint regulator (NCR) targeting improves survival in preclinical sepsis models, but success has been limited in clinical trials (9–15). Strategic targeting of non-redundant NCR pathways has the potential to cater therapeutics to the individual septic patient and improve prognosis (16–18).

B7-CD28 superfamily member V-domain Immunoglobulin Suppressor of T cell Activation (VISTA) is an ideal candidate for such potential strategic targeting in sepsis (18, 19). VISTA is a 55–65-kDa type 1 transmembrane protein and has unique biology that set it apart from all other NCRs (20, 21).

VISTA can act as a receptor or a ligand binding in VISTA : VISTA interactions, with VSIG-3, or with PSGL-1 depending on the cell it is expressed on (22, 23). VISTA regulation is also temporally distinct, acting as the earliest NCR of peripheral tolerance. Under steady-state conditions, VISTA promotes quiescence of naïve CD4^+^ T cells to prevent self-reactivity (24). Under inflammatory conditions, VISTA suppresses effector CD4^+^ T cell function (17, 20), maintains the T regulatory cell (T_reg_) pool size, and promotes induced T_reg_ (iT_reg_) generation (25). This CD4^+^ T cell-specific modularity makes VISTA a specific and non-redundant regulator of the acute T cell response (24–27).

Septic patients experience a reduced number/frequency of splenic and thymic T cells, decreased cytokine production, and increased expression of exhaustion markers (28, 29). In murine sepsis models, there is a significant loss of CD4^+^ T cell frequency which impacts survival (30). Our laboratory, among others, has demonstrated that T_regs_ play an indispensable role in the acute septic response, resolving inflammatory tissue damage and improving survival (31–34).

Based on the findings from our laboratory and others, we set out to determine if the immune checkpoint regulator VISTA controls T-regulatory cells (T_reg_), in response to septic challenge, thus playing a protective role and reducing septic morbidity/mortality. Further, we investigated if changes in morbidity/mortality were due to a T_reg_-mediated effect during the acute response to a septic challenge.

## 2 Material and Methods

### 2.1 Mice

Male C57BL/6 mice were purchased from Jackson Laboratories (Bar Harbor, ME, USA). Animals obtained from our outside vendor were acclimated no less than 7 days, and often longer [maximum ~5 weeks], prior to utilizing these animals in the studies described here. During this period, they were housed in the Rhode Island Hospital (RIH) rodent facility (12-h: 12-h light/dark cycle, 23°C–25°C, 30%–70% humidity) where they received standard care and diet (standard rodent chow)/water *ad libitum*. All protocols were carried out in the morning (8–11 a.m.) and were performed in accordance with the National Institutes of Health guidelines and as approved by the Animal Use Committee of Rhode Island Hospital (AWC# 5064-18 & 5054-21). VISTA^-/-^ mice were produced at the Brown University Transgenic Facility using CRISPR/Cas9 technology. Guide RNA sequences for the 5′ deletion site:

395_Vsir_ex2upsgRNA1: CTTAGTAACAAGACCCACAT

396_Vsir_ex2upsgRNA2: GCTTAGTAACAAGACCCACA

Guide RNA sequences for the 3′ deletion site:

398_Vsir_ex7sgRNA1: ATGTGCACTTGATCTATGGC (18-mer)

399_Vsir_ex7sgRNA2: GTGCCTAAAAGACTGTCCAA

The initial genotyping strategy and PCR results for G1 and F0 generations are described in **Supplemental Figure 1**. A routine genotyping of VISTA^-/-^ mice was performed on tail biopsy samples collected after weaning. Tail samples were processed for PCR and treated with custom 25-nmol DNA oligos from Integrated DNA Technologies (Coralville, IA, USA). Following PCR amplification, samples were run on an SDS-Page gel and imaged for gene deletion analysis and validation. Male mice with appropriate base-pair deletion were used for downstream studies. All mice were housed, bred, and maintained at the Rhode Island Hospital Central Research Facilities.

### 2.2 Patients

Septic/critically ill patients who were admitted to trauma and surgical intensive care units, between July of 2018 and February of 2020, were enrolled in this study per institutional review board approval at Rhode Island Hospital (IRB study # 413013). Inclusion criteria for the study were trauma or sepsis-related critical illness requiring ICU admission. Patients were excluded from the study if they were pregnant or had previous lymphoma or leukemia diagnosis. Patient demographics from the day of blood draw were used to calculate the Acute Physiology of Chronic Health Evaluation II (APACHE II) score (**Table 1**). Healthy volunteers (age- and sex-matched) were enrolled in this study to serve as the control group.

**Table 1:** Patient demographics.

	Healthy Controls	Patients	p-value
Number	8	8	–
Age	48.5 +/- 17.2	58.8 +/- 16.6	0.25
Male gender	5 (62.5%)	6 (75%)	0.62
WBC	–	10.8 +/- 5.0 × 10^6^/ml	–
APACHE II score	–	19.9 +/- 5.2	–
Mortality	–	2 (25%)	–
Active infection	–	7 (87.5%)	–

### 2.3 CLP Model

**Figure 1:**
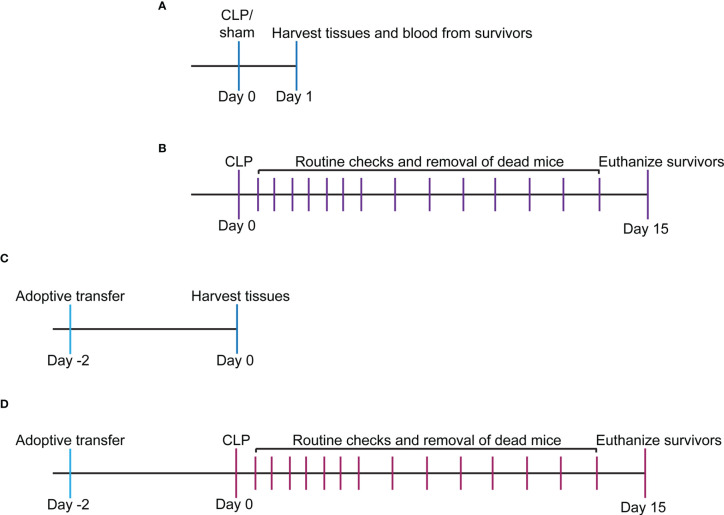
Experimental timeline for study. **(A)** WT and VISTA^-/-^ mice underwent sham or CLP procedure, and tissues/blood were harvested for downstream analysis *via* flow cytometry or spectrophotometry. **(B)** WT and VISTA^-/-^ mice underwent a CLP procedure, and survival was tallied for 14 days. Surviving mice were euthanized on the 15th day. **(C)** WT mice were injected with Jurkat T_regs_, and tissues were harvested 2 days postinjection for downstream analysis and validation of adoptive transfer *via* flow cytometry. **(D)** VISTA^-/-^ mice were injected with Jurkat T_regs_ then underwent CLP 2 days postinjection, and survival was tallied for 14 days. Surviving mice were euthanized on the 15th day.

Cecal ligation and puncture (CLP) as described previously (35–37) was performed on wild-type C57BL/6 and VISTA^-/-^ male mice aged 8–10 weeks. Following midline laparotomy, the cecum was ligated ~1 cm above the cecal tip and punctured twice with a 22-G needle. Cecal contents were extruded into the intraperitoneal cavity. The abdomen was closed using a sterile PDO suture. Mice were treated with lidocaine on the muscle layer and a subcutaneous injection of 1 ml Lactated Ringer’s solution. The choice of male animals was made to maximize our ability to initially see an experimental difference septic response based on previous reports that male mice did poorer in response to these experimental stressors of septic (CLP) challenge than pro-estrus stratified female mice (38, 39). Mice were euthanized 24 h post procedure (based on the experiment as described in **Figure 1**), and tissues were harvested for downstream studies.

### 2.4 Flow Cytometry

#### 2.4.1 Mouse Cell Phenotyping

The spleen, thymus, and intestine were harvested from mice 24 h following sham or CLP procedure. The spleen and thymus tissues were homogenized using frosted slides, and red blood cells were lysed using a Na^+^Cl^-^ gradient. Small intestinal tissue was processed using the Lamina Propria Dissociation Kit (Miltenyi Biotec, Bergisch Gladbach, Germany: cat# 130-097-410) according to the manufacturer protocol. The total cell number from each sample was assessed using Trypan blue stain and hemacytometer counting at ×10 magnification. Samples were diluted to 10^6^ cells/ml in FACS buffer (2 mM EDTA, 0.5% BSA, PBS), Fc blocked, and stained with the following monoclonal anti-mouse antibodies: CD4-BV421 (BioLegend, San Diego, CA, USA, Cat# 100438, RRID : AB_11203718), CD8a-BV510 (BioLegend Cat# 100752, RRID : AB_2563057), CD69-FITC (Miltenyi Biotec Cat# 130-103-950, RRID : AB_2659081), PD-1H/VISTA-PE (BioLegend Cat# 143708, RRID : AB_11150599), CD25-PE/Cyanine7 (BioLegend Cat# 101916, RRID : AB_2616762), and CD152/CTLA-4-PerCP/Cyanine5.5 (BioLegend Cat# 106316, RRID : AB_2564474). Following initial staining, cells were fixed using 4% paraformaldehyde and permeabilized using the True Nuclear Transcription Buffer Set (BioLegend: cat# 424401) according to the manufacturer’s protocol. Permeabilized cells were stained with anti-mouse FOXP3-Alexa Fluor 647 (BioLegend: cat# 126408). To compensate for spectral overlap, UltraComp eBeads Plus Compensation Beads (Thermo Fisher Scientific, Waltham, MA, USA: cat# 01-3333-41) were used according to the manufacturer’s protocol. Fluorescence minus one (FMO) controls were used to determine positive expression gates during analysis using FlowJo software.

#### 2.4.2 Human Cell Phenotyping

Whole blood was drawn from patients and healthy controls, collected in heparin-treated tubes, treated with Ficoll Histopaque-1077, and centrifuged to isolate leukocytes. The leukocyte layer was isolated, washed with PBS, and centrifuged. Cells were counted using a hemacytometer and Trypan blue then diluted to 10^6^ cells/ml in FACS buffer (2 mM EDTA, 0.5% BSA, PBS). Cells were Fc blocked and stained with the following monoclonal anti-human antibodies: CD3-VioBlue (Miltenyi Biotec: Cat# 130-113-133, RRID : AB_2725961) and VISTA-APC (Thermo Fisher Scientific: Cat# 17-1088-42, RRID : AB_2744704). Fluorescence minus one (FMO) control was used to determine positive expression gates during analysis using FlowJo software.

#### 2.4.3 Adoptive Transfer Validation

For adoptive transfer, the pMSCV-mouse Foxp3-EF1α-GFP-T2A-puro stable Jurkat cell line (System Biosciences, Palo Alto, CA, USA: cat# TCL110C-1), referred to as Jurkat T_regs_, was harvested from culture, pelleted *via* centrifugation, and resuspended in HBSS (Thermo Fisher: cat**#** 24020117) at 2 × 10^6^ cells/400 µl. 400 µl of Jurkat T_reg_ suspension or HBSS vehicle control was loaded into a syringe and administered to mouse *via* intraperitoneal injection. Spleen, thymus, and small intestine samples were harvested 48 h post adoptive transfer and processed as described in the previous section. Cells were stained with CD4-BV421 (BioLegend: Cat# 100438, RRID : AB_11203718) and VISTA/PD-1H-APC (BioLegend: Cat# 143709, RRID : AB_11219607). A FMO control was used to determine VISTA-positive expression gates during analysis using FlowJo software.

### 2.5 Colorimetric Assays for Morbidity Study

To assess indices of tissue injury, blood was collected from mice 24 h following sham or CLP procedure *via* cardiac puncture using a heparin-coated syringe. Blood sample was centrifuged at 10,000 rpm, and supernatant (plasma) was collected and stored at -80°C. For tissue injury assays, plasma was analyzed using the following kits according to the manufacturer’s protocol: Urea Nitrogen (BUN) Colorimetric Detection Kit (Invitrogen, Carlsbad, CA, USA, cat# EIABUN), Creatine Kinase Activity Assay Kit (Sigma-Aldrich, St. Louis, MO, USA: cat# MAK116), Alanine Aminotransferase (ALT) Activity Assay Kit (Sigma-Aldrich: cat# MAK052), Aspartate Aminotransferase (AST) Activity Assay Kit (Sigma-Aldrich: cat# MAK055), Amylase Assay Kit (Colorimetric) (Abcam, Cambridge, MA, USA: cat# ab102523), and Bilirubin Assay Kit (Direct Colorimetric) (Abcam: cat# ab235627).

### 2.6 Multiplex Cytokine Analysis

Plasma samples were collected and stored as described in the previous section. To assess the cytokine concentration in plasma samples, the following multiplex kits were used according to the manufacturer’s instruction: LEGENDplex Mouse Inflammation Panel (13-plex) with a V-bottom plate (BioLegend, cat# 740446) and LEGENDplex MU Th Cytokine Panel (12-plex) with VbP VO3 (BioLegend, cat# 741044). Multiplex experiments were carried out using MACSQuant Analyzer 10 (Miltenyi Biotec). Data were analyzed using the LEGENDplex software suite (BioLegend).

### 2.7 *In Vitro* Viability Assay

Jurkat T_regs_ were cultured in RPMI complete medium with 13F3 (Bio X Cell, Lebanon, NH, USA, Cat# BE0310, RRID : AB_2736990) or Ig control (Bio X Cell Cat# BE0091, RRID : AB_1107773) for 30 min at 37°C, 5% CO_2_ then stained with alamarBlue (Bio-Rad, Hercules, CA, USA: product code BUF012A) according to the manufacturer’s protocol. Sample absorbance was measured every 24 h for 7 days using the Bio-Rad spectrophotometer. Viability was calculated according to the manufacturer’s protocol.

### 2.8 *In Vitro* Cytokine Analysis

Jurkat T_regs_ were cultured in RPMI complete medium with 13F3 (Bio X Cell Cat# BE0310, RRID : AB_2736990) or Ig control (Bio X Cell Cat# BE0091, RRID : AB_1107773) overnight at 37°C, 5% CO_2_. Treated cells were then stimulated with 5 µl of plasma from CLP mouse for 2 h prior to harvest from culture. Cells were centrifuged, and supernatant was collected for multiplex analysis using LEGENDplex MU Th Cytokine Panel (12-plex) with VbP VO3 (BioLegend: cat# 741044) according to the manufacturer’s protocol. Multiplex experiments were carried out using MACSQuant Analyzer 10 (Miltenyi Biotec). Data were analyzed using LEGENDplex software suite (BioLegend).

### 2.9 Statistical Analysis

Statistical significance between two groups was determined using either a two-tailed Student’s unpaired *t* test for parametric data or the Mann–Whitney U test for the non-parametric test. Statistical significance between multiple groups was determined using either an ordinary one-way ANOVA for parametric data or the Kruskal–Wallis test for non-parametric data. Alpha was set to 0.05 as the cutoff for statistical significance using Prism 9.3.0 (GraphPad Software) statistical software.

## 3 Results

### 3.1 VISTA Expression Inversely Correlates With T-Cell Population Abundance in Septic Mice and Critically Ill Patients

**Figure 2:**
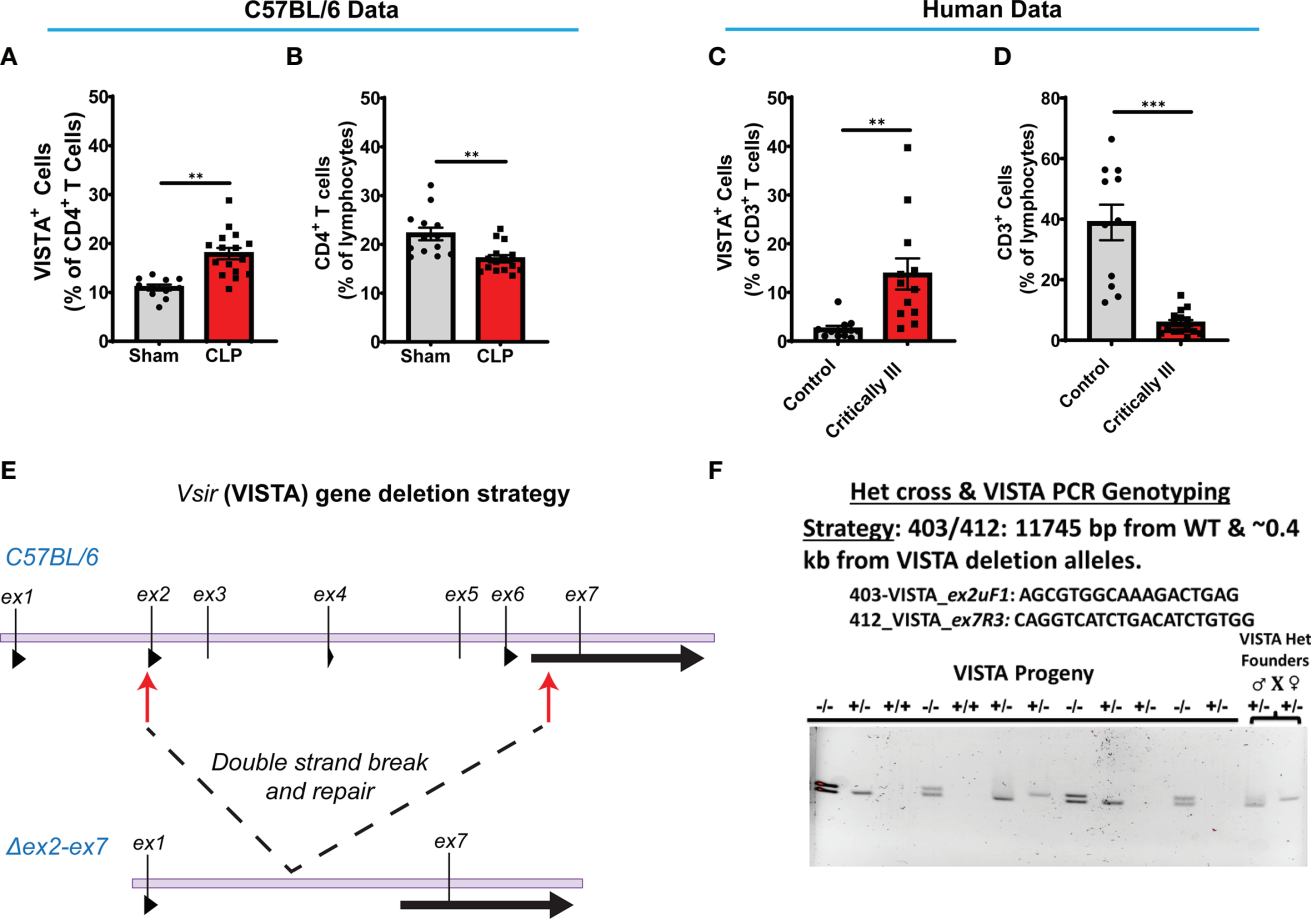
VISTA^+^CD4^+^ T cells in mouse splenocytes and VISTA^+^CD3^+^ lymphocytes in patient blood increase following experimental or clinical sepsis. **(A)** Summary graph of VISTA^+^ CD4^+^ T cells in the wild-type mouse spleen. **(B)** Summary graph of CD4^+^ T cell frequency in the wild-type mouse spleen. **(C)** Summary graph of VISTA^+^ CD3^+^ T cell frequency in the peripheral blood lymphocytes. **(D)** Summary graph of CD3+ T cell frequency in the peripheral blood. **(A–D)** Summary graphs show mean ± SEM [WT-sham: *n = 13*, WT-CLP: *n = 16*]; significance **p < 0.01, ***p < 0.001. **(E)** Initial process (Strategy) for producing embryos deficient in the ~11.3-kb region containing exon (ex) 2 to exon 7 of the VISTA gene on mouse chromosome 10 with CRISPR/Cas9 followed by NHEJ-mediated repair. **(F)** Results of initial heterozygous cross of VISTA^-/+^ founder mice resulting from CRISPR/Cas9 technology that produced homozygous VISTA^-/-^ mice for breeding (PCR genotyping strategy: 403/412: 11,745 bp from WT and ~0.4 kb from VISTA deletion alleles).

Several research groups have shown that during acute sepsis progression there is a significant loss in T cell abundance in the spleen and thymus in both the murine CLP model (29, 40–42) and septic patients (43–46). In this study, we found that C57BL/6 wild-type (WT) mice exhibited a higher VISTA expression on CD4^+^ T cells (**Figure 2A**) and reduced CD4^+^ T cell population abundance (**Figure 2B**) in the spleen following septic challenge.

We enrolled a total of 8 critically ill patients from the trauma and surgical ICUs at a single level-1 trauma center. There was no significant difference between patients and healthy controls regarding gender or age. 87.5% of patients had an active ongoing source of infection at the time of draw, 62.5% required mechanical ventilation, and 37.5% were actively on vasopressor at the time of draw. 25% required dialysis due to critical illness. The average APACHE II score for the population was 19.9 (**Table 1**). Sources of infection included necrotizing soft tissue infections of the lower extremities, intra-abdominal abscesses after perforated hollow viscus injuries, and bacteremia. 75% of enrolled patients met systemic inflammatory response syndrome (SIRS) criteria, 63% met sepsis criteria, and 38% met septic shock criteria (47).

We found that critically ill patients experience a higher VISTA expression on circulating CD3^+^ T cells (**Figure 2C**) despite reduced CD3^+^ T cell population abundance (**Figure 2D**) in circulation. These results suggest that the relationship between VISTA expression and T-cell abundance observed in our murine model of sepsis appear to have a potential correlate in the critically ill septic patient. To further explore the role of VISTA in the sepsis-induced T-cell response and better understand its potential contribution to septic morbidity, we created a global VISTA gene-deficient (VISTA^-/-^) mouse strain using CRISPR/Cas9 technology that could be examined to address this question (**Figures 2E, F**).

Le Tulzo et al. found that T cells become polarized into functionally distinct helper T-cell subsets in sepsis (44), and it is well documented that the regulatory T-cell (T_reg_) subset increases during the acute septic response (46, 48, 49). In light of this, we chose to initially determine how VISTA impacted sepsis-induced T_reg_ polarization by comparing the CD4^+^Foxp3^+^ T_reg_ populations in WT as opposed to VISTA^-/-^ mice *via* flow cytometry (**Supplementary Figure 2**).

### 3.2 CD4^+^ T_reg_ Abundance Increases Following Septic Challenge, But the CD4^+^ T_reg_ Population Is Significantly Smaller in Peripheral T-Cell Compartments of VISTA^-/-^ Mice Compared to WT Mice

**Figure 3:**
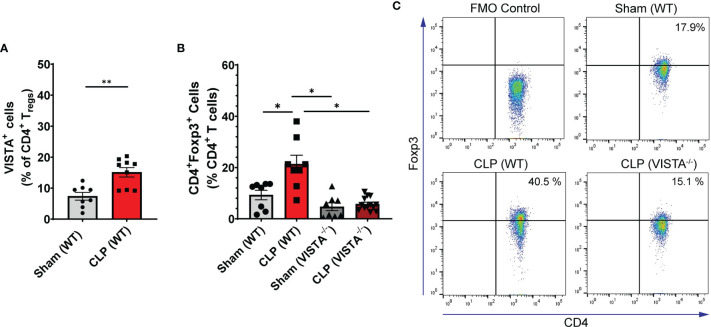
VISTA expression correlates with CD4^+^ T_reg_ population increase following CLP and VISTA^-/-^ mice fail to expand the CD4^+^T_reg_ population in the spleen. **(A)** Summary graph of VISTA^+^ CD4^+^ T_reg_ frequency in the spleen. **(B)** Summary graph of CD4^+^ T_reg_ frequency in the spleen and **(C)** representative flow cytometry plots comparing fluorescence minus one (FMO) control, sham (WT), CLP (WT), and CLP (VISTA^-/-^) samples. Summary graphs show mean ± SEM [WT-sham: *n = 8*, WT-CLP: *n = 8*, VISTA^-/^ **^–^** sham: *n = 8*, VISTA^-/-^ -CLP: *n = 13*]; significance *p < 0.05; **p < 0.01.

**Figure 4:**
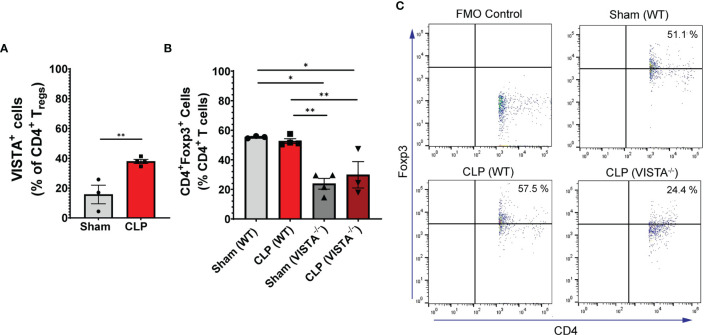
VISTA expression correlates with CD4^+^ T_reg_ population increase following CLP and VISTA^-/-^ mice fail to expand the CD4^+^ T_reg_ population in the intestinal intraepithelial compartment. **(A)** Summary graph of VISTA^+^ CD4^+^ T_reg_ frequency in the small intestine. **(B)** Summary graph of CD4^+^ T_reg_ frequency in the small intestine and **(C)** representative flow cytometry plots comparing fluorescence minus one (FMO) control, sham (WT), CLP (WT), and CLP (VISTA^-/-^) samples. Summary graphs show mean ± SEM [WT-sham: *n = 3*, WT-CLP: *n = 3*, VISTA^-/-^sham: *n = 4*, VISTA^-/-^ -CLP: *n = 4*]; significance *p < 0.05; **p < 0.01.

We found that in the WT spleen, there is a significant increase in total proportion of CD4^+^ T_regs_ and VISTA^+^CD4^+^ T_regs_ following CLP (**Figures 3A–C**). VISTA^-/-^ mice exhibit decreased abundance of total CD4^+^ T_regs_ in the spleen (**Figures 3B, C**). In the thymus, we observe no change in VISTA expression on the T_reg_ populations between sham and CLP WT mice (**Supplementary Figures 3A, D**); however, VISTA^-/-^ mice have higher total abundance of CD4^+^ T_regs_ and CD4^+^CD8^+^ T_regs_ compared to WT mice (**Supplementary Figures 3B, E**). In the intraepithelial compartment of the small intestine, the frequency of VISTA^+^CD4^+^ T_regs_ increases significantly following CLP (**Figure 4A**) and VISTA^-/-^ mice have less CD4^+^ T_regs_ under steady-state (sham) and inflammatory (CLP) conditions compared to WT mice (**Figures 4B, C**). We did not observe any trends in the lamina propria compartment of the small intestine (**Supplementary Figure 4**).

### 3.3 CD4^+^ T_regs_ Demonstrate Compensatory Upregulation of Several Checkpoint Proteins and Suppressive Factors in VISTA^-/-^ Mice

**Figure 5:**
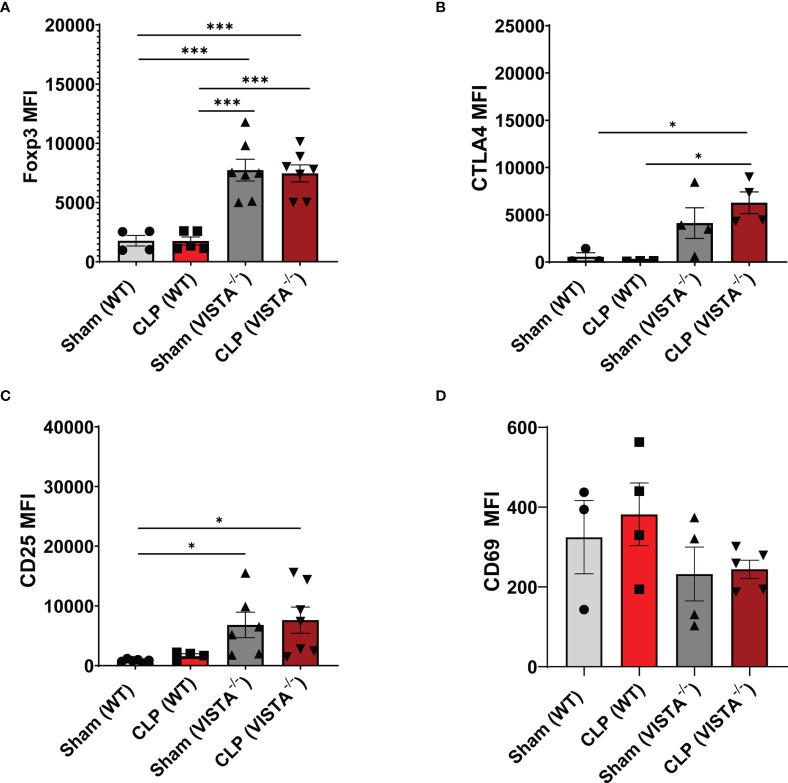
Expression of suppressive markers is upregulated on CD4^+^ T_regs_ in the spleen of VISTA^-/-^ mice. Median fluorescence intensity (MFI) of **(A)** Foxp3, **(B)** CTLA4, **(C)** CD25, and **(D)** CD69 on CD4^+^ T_regs_ in the spleen. Summary graphs show mean ± SEM [WT-sham: *n = 3*, WT-CLP: *n = 3*, VISTA^-/-^sham: *n = 4*, VISTA^-/-^ -CLP: *n = 4*]; significance *p < 0.05; ***p < 0.001.

**Figure 6:**
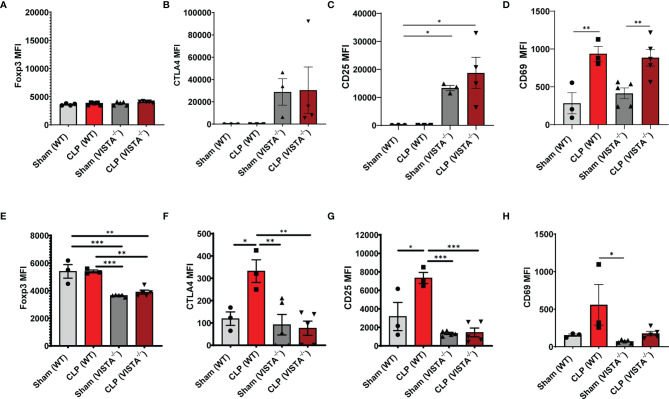
Expression of suppressive markers is upregulated on CD4^+^ T_regs_ and is downregulated on CD4^+^CD8^+^ T_regs_ in the thymus of VISTA^-/-^ mice. Median fluorescence intensity (MFI) of **(A)** Foxp3, **(B)** CTLA4, **(C)** CD25, and **(D)** CD69 on CD4^+^ T_regs_ in the thymus. Median fluorescence intensity (MFI) of **(E)** Foxp3, **(F)** CTLA4, **(G)** CD25, and **(H)** CD69 on CD4^+^CD8^+^ T_regs_ in the thymus. Summary graphs show mean ± SEM [WT-sham: *n = 3*, WT-CLP: *n = 3*, VISTA^-/-^sham: *n = 4*, VISTA^-/-^ -CLP: *n = 4*]; significance *p < 0.05; **p < 0.01; ***p < 0.001.

The loss in CD4^+^ T_regs_ in VISTA^-/-^ mice lead us to ask if the cell-surface expression signature, as it related to suppressive function of these cells, was altered by CLP. In the spleen, Foxp3, CTLA4, and CD25, but not CD69, are significantly upregulated on CD4^+^ T_regs_ in VISTA^-/-^ mice compared to WT mice under steady-state and inflammatory conditions (**Figures 5A–D**). In the thymus (**Figures 6A–G**), CD25 is significantly upregulated on CD4^+^ T_regs_ in VISTA^-/-^ mice compared to WT mice under steady state and inflammatory conditions (**Figure 6C**). CD4^+^ T_regs_ upregulate CD69 following CLP in WT and VISTA^-/-^ mice (**Figure 6D**). We also found that Foxp3, CTLA4, and CD25 are significantly upregulated on CD4^+^CD8^+^ T_regs_ in WT mice compared to VISTA^-/-^ mice under steady-state and inflammatory conditions (**Figures 6E–G**). In the lamina propria compartment, we observe a significant upregulation of CTLA4 on CD4^+^ T_regs_ in VISTA^-/-^ mice (**Supplementary Figure 5B**). However, this trend is not observed in the small intestinal intraepithelial compartment (**Supplementary Figure 6**).

### 3.4 VISTA^-/-^ Mice Have Higher Th17-Related Cytokine Production Compared to WT Mice Following Septic Challenge

**Figure 7:**
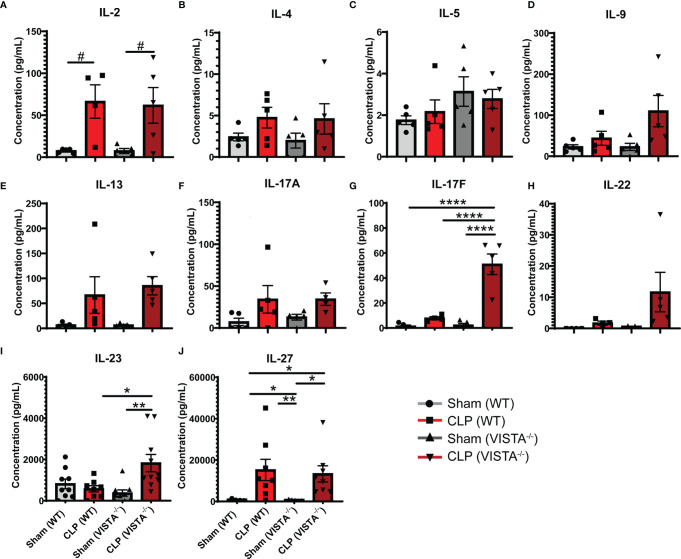
VISTA^-/-^ mice have significantly higher levels of several Th17 cytokines following septic challenge. **(A–J)** Plasma cytokine concentration of wild-type and VISTA^-/-^ mice. Summary graphs show mean ± SEM [WT-sham: *n = 5*, WT-CLP: *n = 5*, VISTA^-/-^sham: *n = 5*, VISTA^-/-^ -CLP: *n=5*]; significance ^#^p = 0.05; *p < 0.05; **p < 0.01; ****p < 0.0001.

To expand from the T_reg_ phenotyping described above, we sought to measure the abundance of several cytokines in circulation implicated in the helper T cell response (**Figures 7A–J**). We found that VISTA^-/-^ mice have significantly higher circulating IL-17F and IL-23 compared to WT mice post CLP (**Figures 7G, I**).

### 3.5 Compensatory Upregulation of Foxp3, CTLA4, and CD25 on Peripheral T_reg_ Populations Correlates With Decreased Survival in VISTA^-/-^ Mice

**Figure 8:**
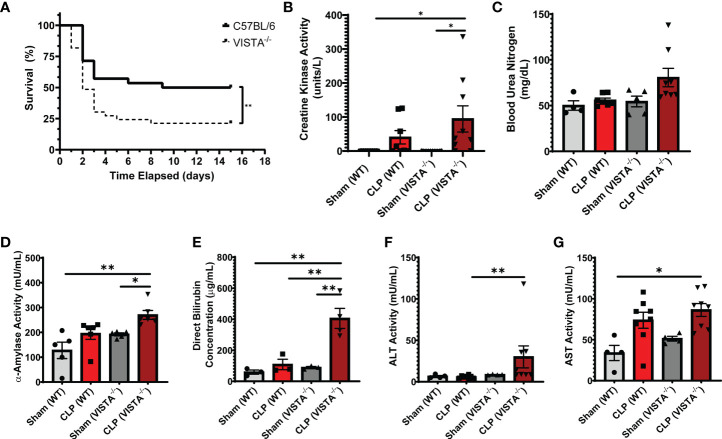
VISTA^-/-^ mice have significantly worse survival and morbidities following septic challenge. **(A)** 14-day survival following CLP [WT: *n = 28*, VISTA^-/-^: *n = 26*]. **(B)** Creatine kinase activity [WT-sham: *n = 9*, WT-CLP: *n = 9*, VISTA^-/-^sham: *n = 10*, VISTA^-/-^ -CLP: *n = 10*]. **(C)** blood urea nitrogen [WT-sham: *n = 4*, WT-CLP: *n = 9*, VISTA^-/-^sham: *n = 5*, VISTA^-/-^ -CLP: *n = 8*]. **(D)** α-Amylase activity [WT-sham: *n = 5*, WT-CLP: *n = 6*, VISTA^-/-^sham: *n = 5*, VISTA^-/-^ -CLP: *n = 6*]. **(E)** Direct bilirubin concentration [WT-sham: *n = 3*, WT-CLP: *n = 3*, VISTA^-/-^sham: *n = 3*, VISTA^-/-^ -CLP: *n = 4*]. **(F)** alanine aminotransferase activity [WT-sham: *n = 4*, WT-CLP: *n = 8*, VISTA^-/-^sham: *n = 4*, VISTA^-/-^ -CLP: *n = 8*]. **(G)** Aspartate aminotransferase activity [WT-sham: *n = 4*, WT-CLP: *n = 8*, VISTA^-/-^sham: *n = 4*, VISTA^-/-^ -CLP: *n = 8*] from plasma samples of wild-type and VISTA^-/-^ mice. **(B–G)** Summary graphs show mean ± SEM; significance *p < 0.05; **p < 0.01.

**Figure 9:**
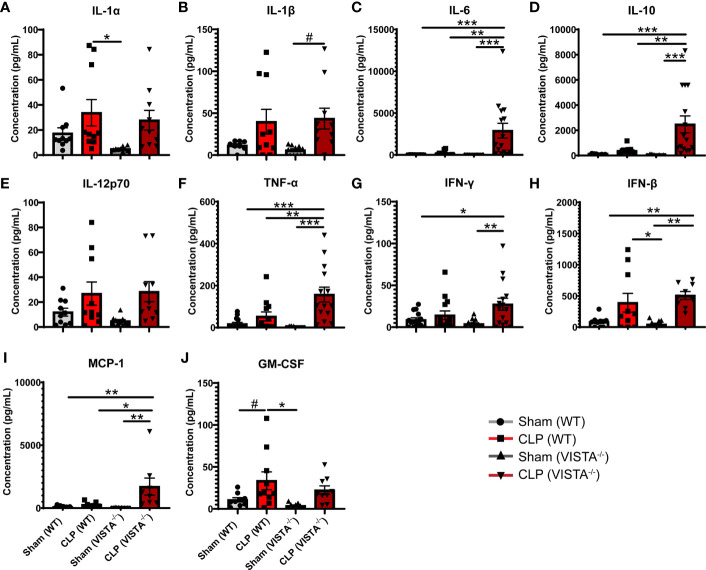
VISTA^-/-^ mice have significantly higher levels of several proinflammatory cytokines following septic challenge. **(A–J)** Plasma cytokine concentration of wild-type and VISTA^-/-^ mice. Summary graphs show mean ± SEM [WT-sham: *n = 10*, WT-CLP: *n = 10*, VISTA^-/-^sham: *n = 10*, VISTA^-/-^ -CLP: *n = 10*]; significance ^#^p = 0.05; *p < 0.05; **p < 0.01; ***p < 0.001.

Based on the apparent compensatory upregulation of suppressive T_reg_ mediators, we decided to compare the mortality and morbidity of VISTA^-/-^ as opposed to WT mice when subjected to CLP (**Figures 8A–J**). We found that VISTA^-/-^ mice had significantly decreased survival, which coincided with increased blood bilirubin, ALT, and AST 24 h following CLP (**Figures 8A, D–G**). VISTA^-/-^ mice also present a more proinflammatory systemic cytokine profile (**Figures 9A–J**). These mice exhibit higher circulating IL-6, IL-10, TNF-α, and MCP-1 compared to WT mice post CLP (**Figures 9C, D, G, I**).

### 3.6 Adoptive Transfer of VISTA-Expressing T_regs_ to VISTA^-/-^ Mice Rescues Survival to WT Levels Following CLP

**Figure 10:**
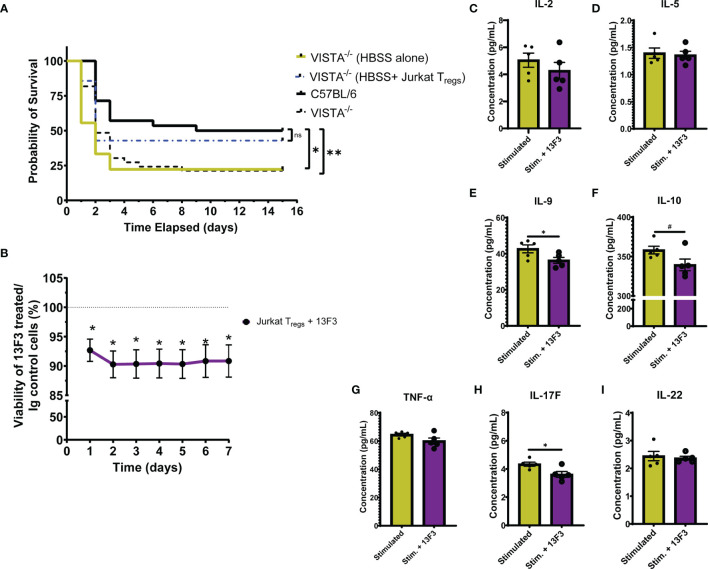
Adoptive transfer of Jurkat T_regs_ improves survival of VISTA^-/-^ mice post CLP. Blockade of VISTA in Jurkat T_regs_ *in vitro* reduces viability and cytokine production. **(A)** 14-day survival following adoptive transfer and CLP [VISTA^-/-^(HBSS alone): *n = 11*, VISTA^-/-^(HBSS+ Jurkat T_regs_): *n = 10*]. **(B)** Alamar Blue viability assay of Jurkat T_regs_ following treatment with Ig control or 13F3. **(C–I)** Supernatant cytokine concentration of Jurkat T_regs_ following treatment with plasma from septic mouse (stimulated) or plasma and 13F3 (stim. + 13F3). Summary graphs show mean ± SEM; not significant ^ns^; significance ^#^p = 0.05; *p < 0.05; **p < 0.01.

To establish the contribution of VISTA^+^ T_regs_ to survival in murine sepsis, we chose to adoptively transfer pMSCV-mouse Foxp3-EF1α-GFP-T2A-puro stable Jurkat cells, hereby referred to as Jurkat T_regs_, into VISTA^-/-^ mice prior to CLP. 48 h post adoptive transfer, Jurkat T_regs_ accumulate in the spleen, thymus, and small intestine (**Supplementary Figures 7A–D**) and express high levels of VISTA (**Supplementary Figures 7E–H**). Based on these results, we performed the adoptive transfer 48 h before CLP and then subsequently assessed overall survival. We found that, following Jurkat T_reg_ adoptive transfer, VISTA^-/-^ mice had comparable survival to WT mice post CLP (**Figure 10A**).

### 3.7 *In Vitro*, VISTA Blockade Reduces Jurkat T_reg_ Viability and Cytokine Production

In addition to establishing the relevance of VISTA-expressing T_regs_ in septic mouse morbidity/mortality, we wanted to uncover how VISTA expression/ligation might be directly impacting T_reg_ function. To do this, we again utilized the Jurkat T_reg_ cell line for mechanistic *in vitro* studies. Jurkat T_regs_ were pretreated with a commercially available VISTA-neutralizing antibody, 13F3, or antibody control then stained with alamarBlue. alamarBlue is a redox indicator used to measure metabolic activity as a readout for viability. The concentration of alamarBlue was assessed *via* a spectrophotometer every 24 h for 7 days. We found that there was a significant reduction in viability following treatment and this reduction was maintained for 7 days without additional 13F3 treatment (**Figure 10B**). Upon *in vitro* acute stimulation of Jurkat T_regs_ with plasma from septic mice, these cells produce several helper T cell-related cytokines (**Figures 10C–I**) but failed to produce IFN-γ, IL-4, or IL-17A (**Supplementary Figure 8**). Interestingly, 13F3-treated Jurkat T_regs_ produce lower levels of IL-9, IL-10, and IL-17F following acute stimulation (**Figures 10E, F, H**).

## 4 Discussion

Since its initial discovery, VISTA has been implicated in diverse immune-related pathologies driven by both innate and adaptive cells (20–27, 50–54). In a preliminary study, we found that septic mice and critically ill patients exhibit a higher proportion of VISTA^+^ T cells as compared to healthy controls. Based on these results, we set out to determine the impact of VISTA expression on regulatory T cells (T_regs_) in murine sepsis. The CD4^+^ T_reg_ plays a vital role in peripheral tolerance, regulation of effector cells, and prevention of bystander tissue damage under inflammatory and steady-state conditions as reviewed by Corthay (55). In sepsis, peripheral T_regs_ increase significantly and correlate with patient outcomes and lymphoproliferative pathology as reviewed by Jiang et al. (56, 57).

### 4.1 The T_reg_ Population Is Composed of Several Subpopulations That Arise From Diverse Stimuli and Developmental Programs

The T_reg_ classification as a distinct T cell lineage has been a point of contention due to the inherent plasticity of T_regs_ and the lack of a definitive “T_reg_” marker as effector T cells can transiently express T_reg_ markers upon activation. Forkhead/winged-helix transcription factor box P3 (Foxp3) is arguably the most reliable T_reg_ marker in mice and was used to delineate effector T cells and T_regs_ in this study (55, 58, 59). CD4^+^Foxp3^+^ T cells are potent suppressors of effector CD4^+^ T cells, CD8^+^ T cells, natural killer (NK) cells, dendritic cells (DCs), and B cells under inflammatory conditions (55).

### 4.2 VISTA Plays a Role in T-Cell Polarizing Cytokine Production and CD4^+^ T_reg_ Abundance in Sepsis

In this study, we found that VISTA expression and total CD4^+^ T_reg_ abundance increase significantly during the acute septic response. Further, this increase in peripheral T_reg_ abundance is dependent on VISTA expression. We also found that VISTA expression plays a role in orchestrating the cytokine response to septic challenge. Cytokines provide contextual immunologic cues that shape cell lineage determination and plasticity. Higher levels of IL-17F, IL-6, and IL-23 promote CD4^+^ T cell polarization toward a Th17 phenotype, and higher concentrations of these cytokines may explain the reduced T_reg_ abundance observed in VISTA^-/-^ mice (60, 61). Previous studies found that VISTA regulated the T_reg_–Th17 polarization axis in mice (25), further supporting our results in the context of sepsis.

### 4.3 VISTA^-/-^ Mice Experience Compensatory Upregulation of Several Endogenous Mediators of T_reg_ Suppression Such as CTLA4, Foxp3, and CD25 Under Steady-State Conditions

Interestingly, CTLA4 expression regulates the turnover and maintenance of T_regs_ at steady state while Foxp3 regulates T_reg_ function and lineage commitment (62–64). A steady-state T_reg_ pool is requisite for preventing autoimmune lymphoproliferative pathology (65). Several groups have shown that VISTA^-/-^ mice do not exhibit overt autoimmune pathologies under tolerogenic conditions (26, 53, 66). Therefore, we posit that the higher baseline expression of CTLA4, Foxp3, and CD25 in VISTA-deficient CD4^+^ T_regs_ represents an inherent compensatory mechanism to sustain peripheral tolerance under tolerogenic conditions.

### 4.4 In the Acute Immune Response to Infection, as Observed With Our Murine Model of Sepsis, Compensatory Upregulation of CTLA4, Foxp3, and CD25 by CD4^+^ T_regs_ Is Insufficient

An explanation may lie in the efficacy of CTLA4, Foxp3, and/or CD25-mediated suppression in our model. CD4^+^ T_regs_ utilize diverse contact-dependent and independent mechanisms to exert immune suppression (35–37, 67, 68). For example, CTLA4-expressing T_regs_ bind to B7-1/2 on antigen-presenting DCs, promoting trans-endocytosis of B7-1/2 and preventing DC-mediated activation of effector T cells. CD25 scavenges IL-2 from the environment, reduces IL-2 activation of effector T cells *via* competitive inhibition, and regulates the function of mature DCs (69).

Another mechanism by which T_regs_ exert immune suppression is by directly polarizing the monocyte lineage commitment from M1 to M2 macrophages (69, 70). M1 macrophages produce proinflammatory cytokines and exacerbate inflammation-derived tissue injury in sepsis (71). Two potent M1 cytokines, IL-6 and MCP-1, are highly upregulated in VISTA^-/-^ mice following septic challenge. M1-mediated pathology is particularly profound in the liver during infection (72), which may explain the increased acute liver injury observed in septic VISTA^-/-^ mice.

### 4.5 Higher M1-Associated Cytokines and Apparent Liver Injury in VISTA^-/-^ Mice Represents a Possible Lapse in the Suppressive Capacity of VISTA^-/-^ T_regs_ Despite Compensatory Upregulation of CTLA4, Foxp3, and CD25

To determine if VISTA^-/-^ T_regs_ contribute to the survival deficit observed in VISTA^-/-^ mice, we adoptively transferred VISTA-overexpressing Jurkat T_regs_ into VISTA^-/-^ mice prior to septic challenge. We found that addition of Jurkat T_regs_ into VISTA^-/-^ mice rescues survival to wild-type levels. Upon VISTA blockade *in vitro*, the Jurkat T_regs_ exhibited reduced proliferative capacity and production of IL-9 and IL-10. T_reg_-derived IL-9 plays a significant role in recruiting other suppressive immune cells, such as mast cells, to suppress bystander tissue damage as observed in a murine nephrotoxic serum nephritis model (73). T_reg_-derived IL-10 is required to regulate effector T cells during acute inflammation (74, 75).

A recent study was published demonstrating a survival benefit upon VISTA antibody blockade prior to CLP (76). Importantly, in the Tao et al. study they utilized WT mice. However, it has also been shown that VISTA-gene-deficient mice have a predisposition to proinflammatory immune activation in several disease contexts (17, 20, 26, 66). Based on prior studies and our results, we believe that the VISTA-gene-deficient mice develop a predisposition to proinflammatory tissue injury that is exacerbated by CLP, thus resulting in a survival deficit. Consequently, acute VISTA blockade with an exogenous antibody in a developmentally normal WT mouse, as used in the Tao et al. study, might yield different results than observed in VISTA^-/-^ mice in our study.

In conclusion, we found that WT mice have increased VISTA^+^CD4^+^ T_regs_ and increased total CD4^+^ T_regs_ in the spleen and small intestine post CLP. This increase in total CD4^+^ T_reg_ abundance is lost in VISTA^-/-^ mice; however, VISTA^-/-^ CD4^+^ T_regs_ have a higher expression of Foxp3, CTLA4, and CD25 relative to WT mice. VISTA^-/-^ mice also have an altered cytokine profile including higher IL-6, IL-10, TNF-a, IL-17F, IL-23, and MCP-1 relative to WT mice. VISTA^-/-^ mice have higher indices of acute liver injury (i.e., bilirubin, ALT, and AST) and reduced survival post CLP compared to WT mice. Interestingly, we were able to rescue VISTA^-/-^ survival to WT levels by adoptively transferring VISTA-expressing Jurkat T_regs_ into VISTA^-/-^ mice prior to CLP. In addition, treating Jurkat T_regs_ with a VISTA-neutralizing antibody *in vitro*, reduced viability and cytokine production. We can conclude from these experiments that VISTA expression plays a pivotal role in promoting acute CD4^+^ T_reg_ survival/stability and regulating the cytokine milieu in acute sepsis to confer a survival benefit.

### 4.6 Future Considerations

This study has raised questions as to the mechanism by which VISTA promotes T_reg_ survival. Interestingly, Foxp3 and VISTA are both under the transcriptional regulation of p53 and HIF-1α. In fact, p53-Foxp3 and HIF1α-Foxp3 induction are indispensable for protective T_reg_ suppression under inflammatory conditions (24, 77–79). The tentative relationship between VISTA and Foxp3 expression provide an additional line of query regarding T_reg_ plasticity.

Another area for further investigation concerns the effector immune cells that are non-redundantly regulated by VISTA^+^ T_regs_. Based on results from this study, VISTA may act as a non-redundant marker for the T_reg_ subset responsible for regulating M1/M2 polarization and limiting acute liver injury in sepsis. More work must be done to fully elucidate these mechanisms; however, we think this study contributes a novel perspective on checkpoint regulator, VISTA, in the acute sepsis response.

## Data Availability Statement

The original contributions presented in the study are included in the article/**Supplementary Material**. Further inquiries can be directed to the corresponding author.

## Ethics Statement

The studies involving human participants were reviewed and approved by institutional review board approval at Rhode Island Hospital (IRB study # 413013). The patients/participants provided their written informed consent to participate in this study. All protocols were performed in accordance with the National Institutes of Health guidelines and as approved by the Animal Use Committee of Rhode Island Hospital (AWC# 5064-18 and 5054-21).

## Author Contributions

CG provided substantial contribution to the conception of this project, experiment design, data acquisition, and data analysis. CG also wrote the initial draft of the manuscript and participated in the revision steps of the manuscript. BB-G provided initial contribution to the conception of this project. MW enrolled patients and healthy controls, collected samples, and acquired human data. C-SC aided in small intestine sample isolation for flow cytometry studies. YC performed the initial survival study of VISTA^-/-^ and WT mice and performed routine genotyping and husbandry of VISTA^-/-^ mice. YQ-R performed initial ELISAs that supported multiplex experiments and results. JT generated preliminary data that contributed to the initial conception of this project. AA provided guidance throughout the conception and execution of this project. All authors reviewed this manuscript. All authors contributed to the article and approved the submitted version.

## Funding

This study was supported by the National Institutes of Health (R35 GM118097 [provided for costs of supplies/animals/reagents, and portions of salary for AA, CG], R25 GM083270 [provided for a portion salary for CG], T32 GM065085 [provided fellowship support for MW], T32 HL134625 [provided fellowship support for BB-G], T35 HL67704 [provided fellowship support for YQ-R, JDT]).

## Conflict of Interest

The authors declare that the research was conducted in the absence of any commercial or financial relationships that could be construed as a potential conflict of interest.

## Publisher’s Note

All claims expressed in this article are solely those of the authors and do not necessarily represent those of their affiliated organizations, or those of the publisher, the editors and the reviewers. Any product that may be evaluated in this article, or claim that may be made by its manufacturer, is not guaranteed or endorsed by the publisher.
