# Supplementary figures and images for: Negative Immune Checkpoint Protein, VISTA, Regulates the CD4+ Treg Population During Sepsis Progression to Promote Acute Sepsis Recovery and Survival
Source: Front Immunol. 2022 Mar 24;13:861670. doi: 10.3389/fimmu.2022.861670 (PMC8988198; doi:10.3389/fimmu.2022.861670)

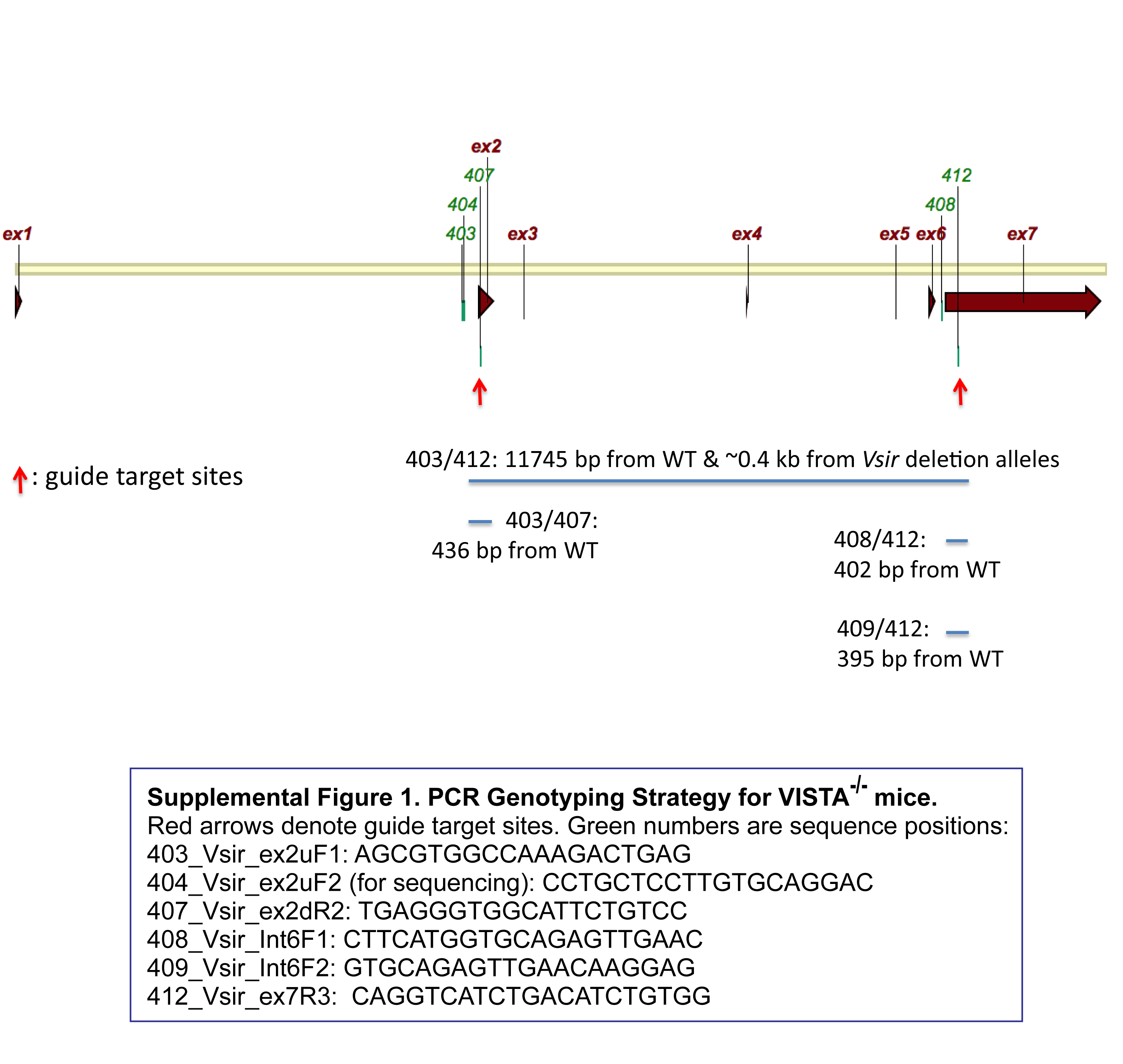

Supplement: Supplementary file 1 [file Image_1.tif]

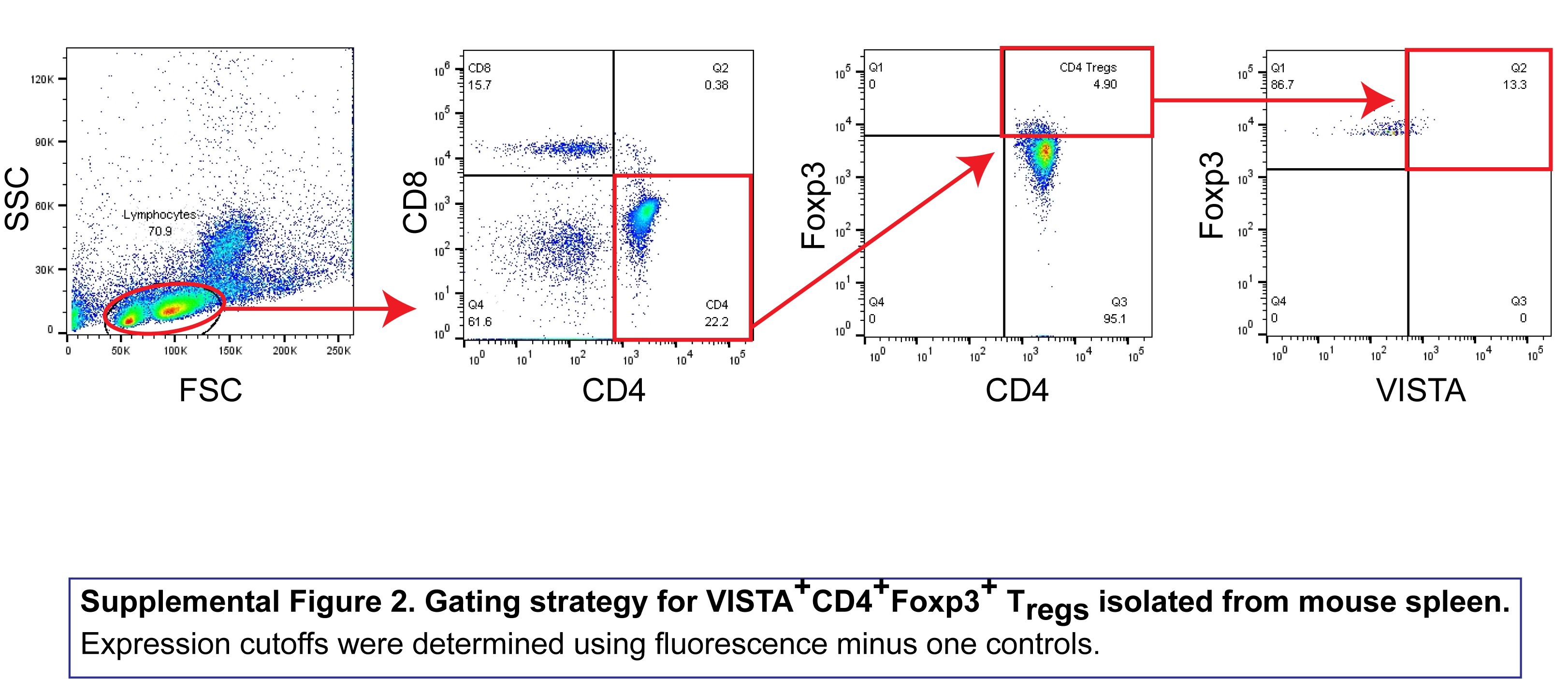

Supplement: Supplementary file 2 [file Image_2.tif]

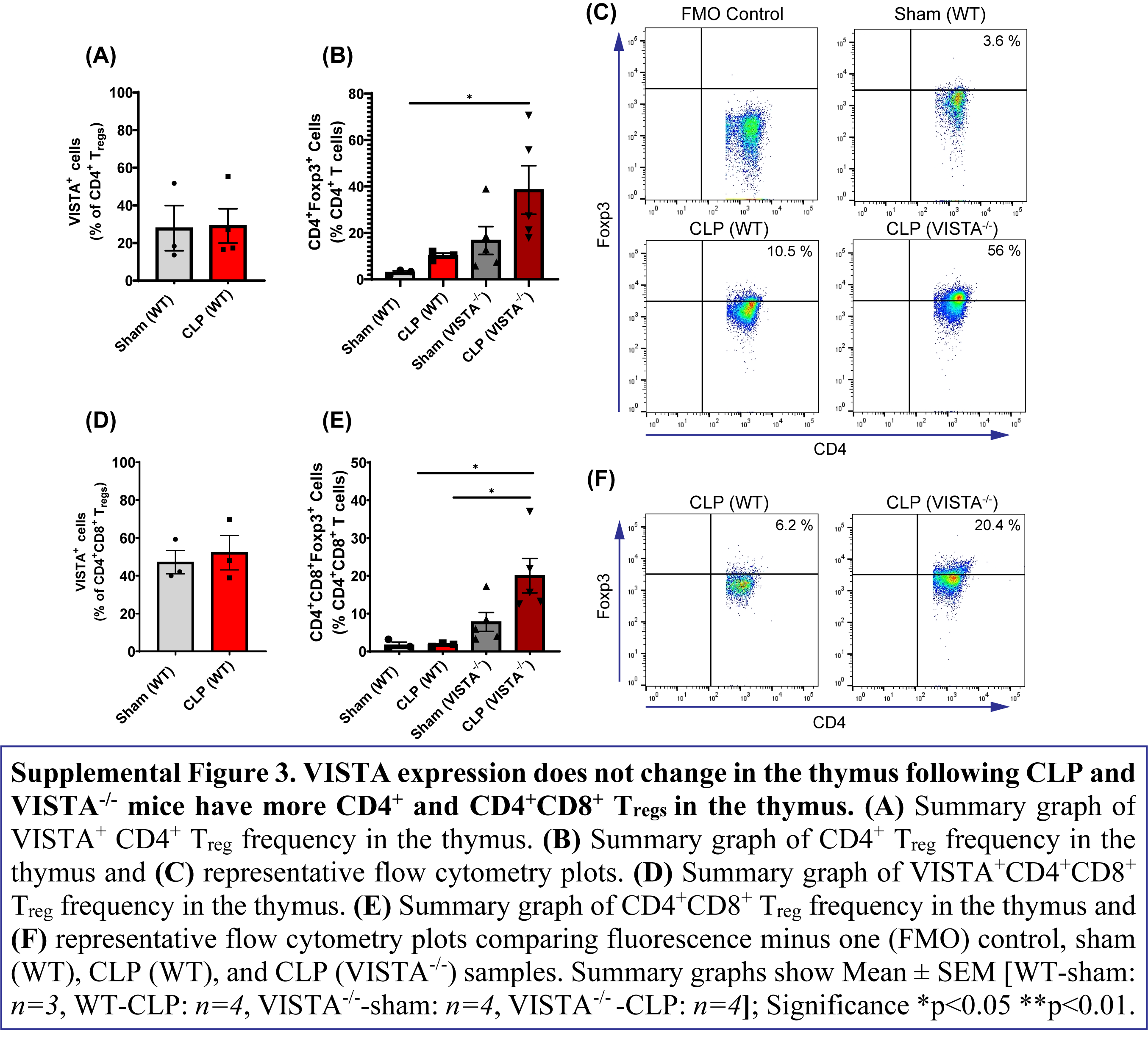

Supplement: Supplementary file 3 [file Image_3.tif]

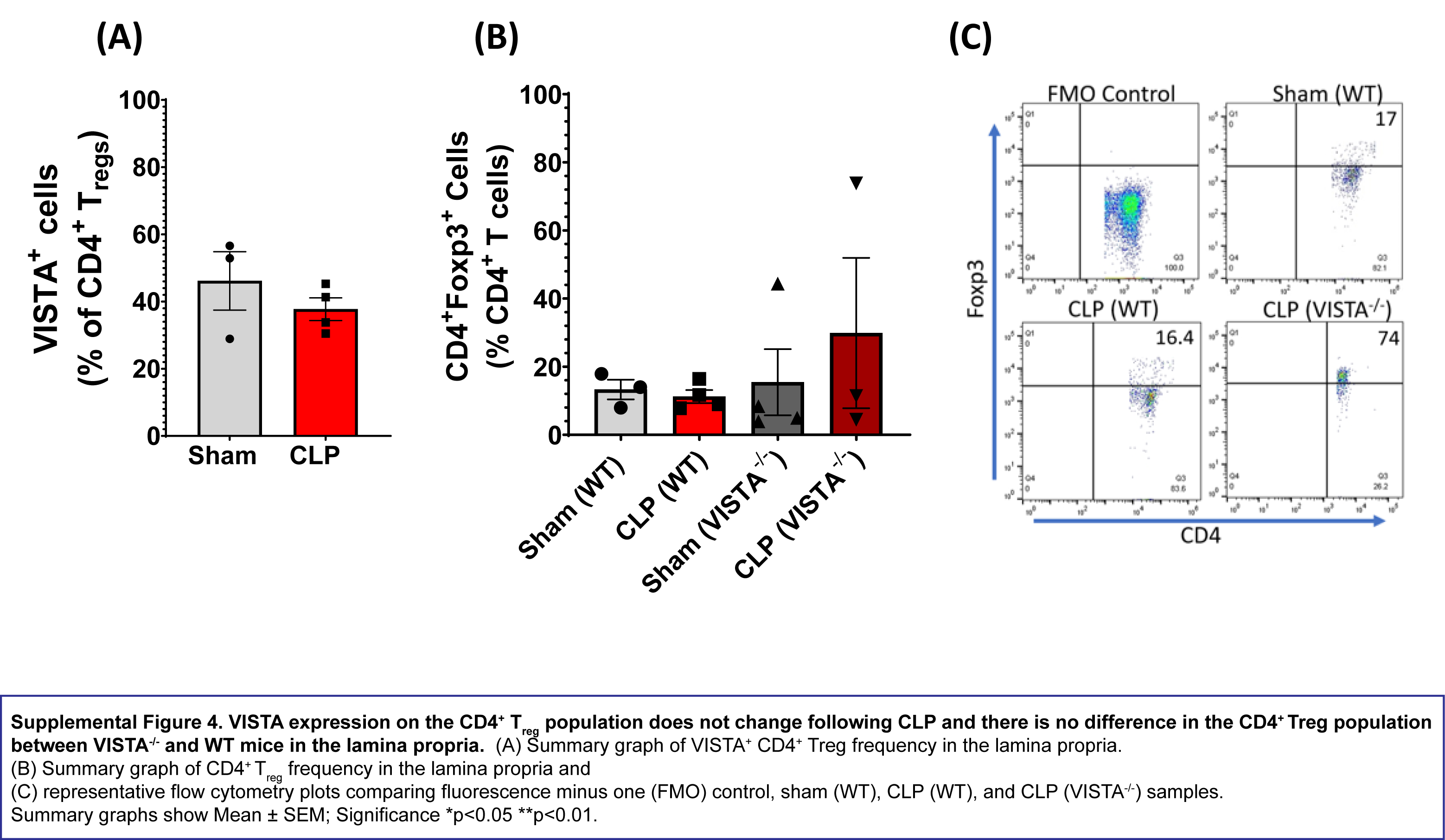

Supplement: Supplementary file 4 [file Image_4.tif]

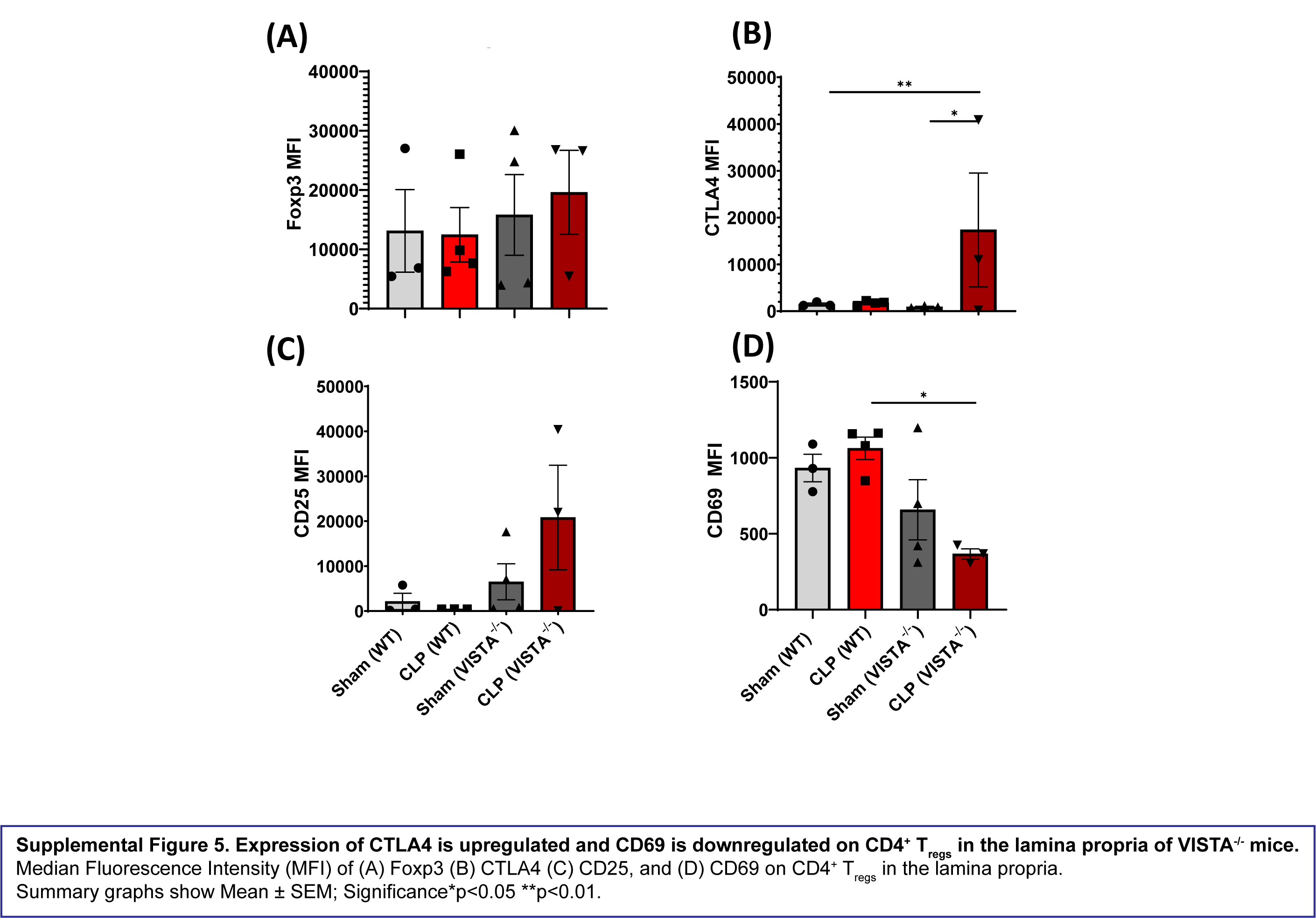

Supplement: Supplementary file 5 [file Image_5.tif]

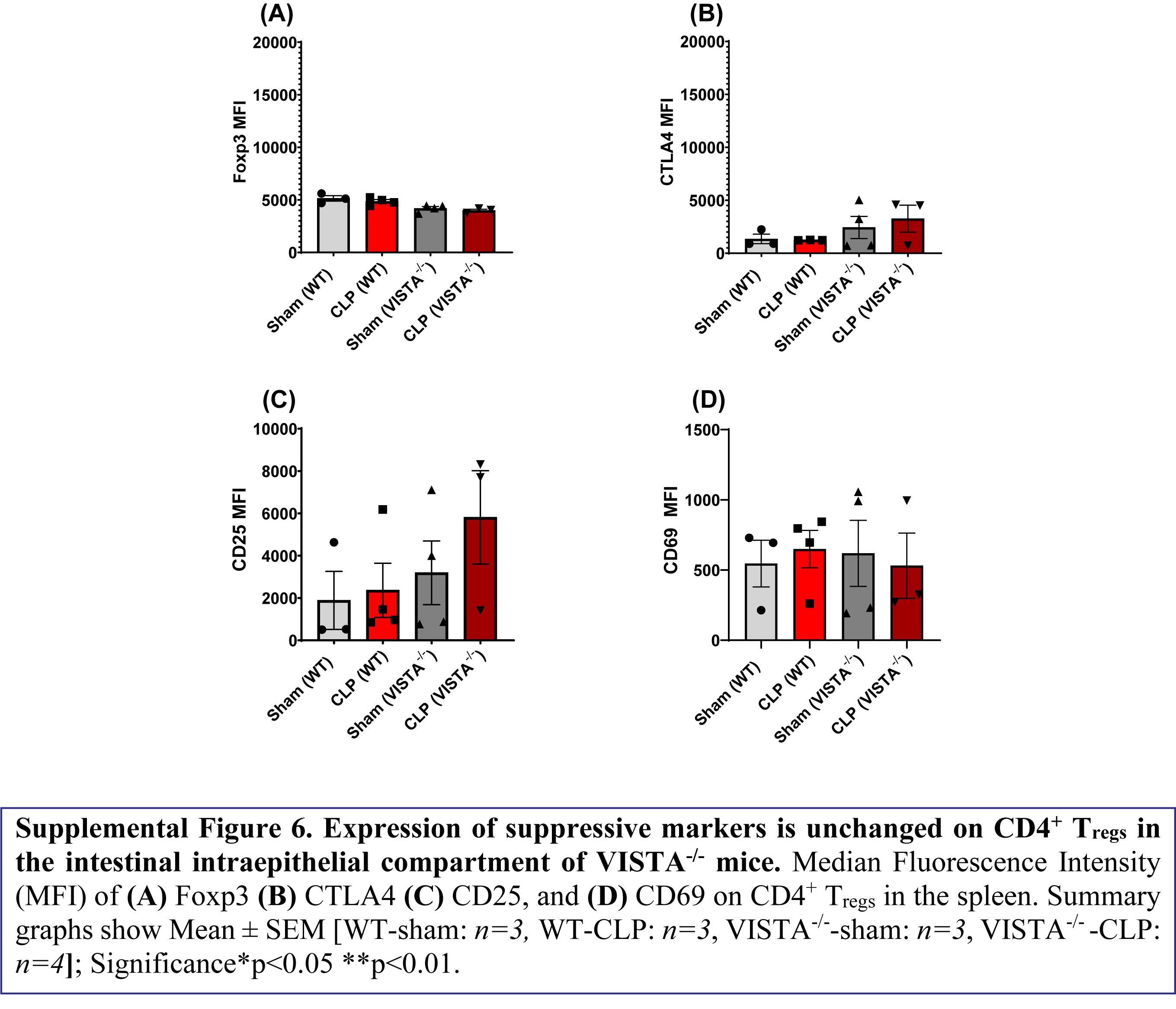

Supplement: Supplementary file 6 [file Image_6.tif]

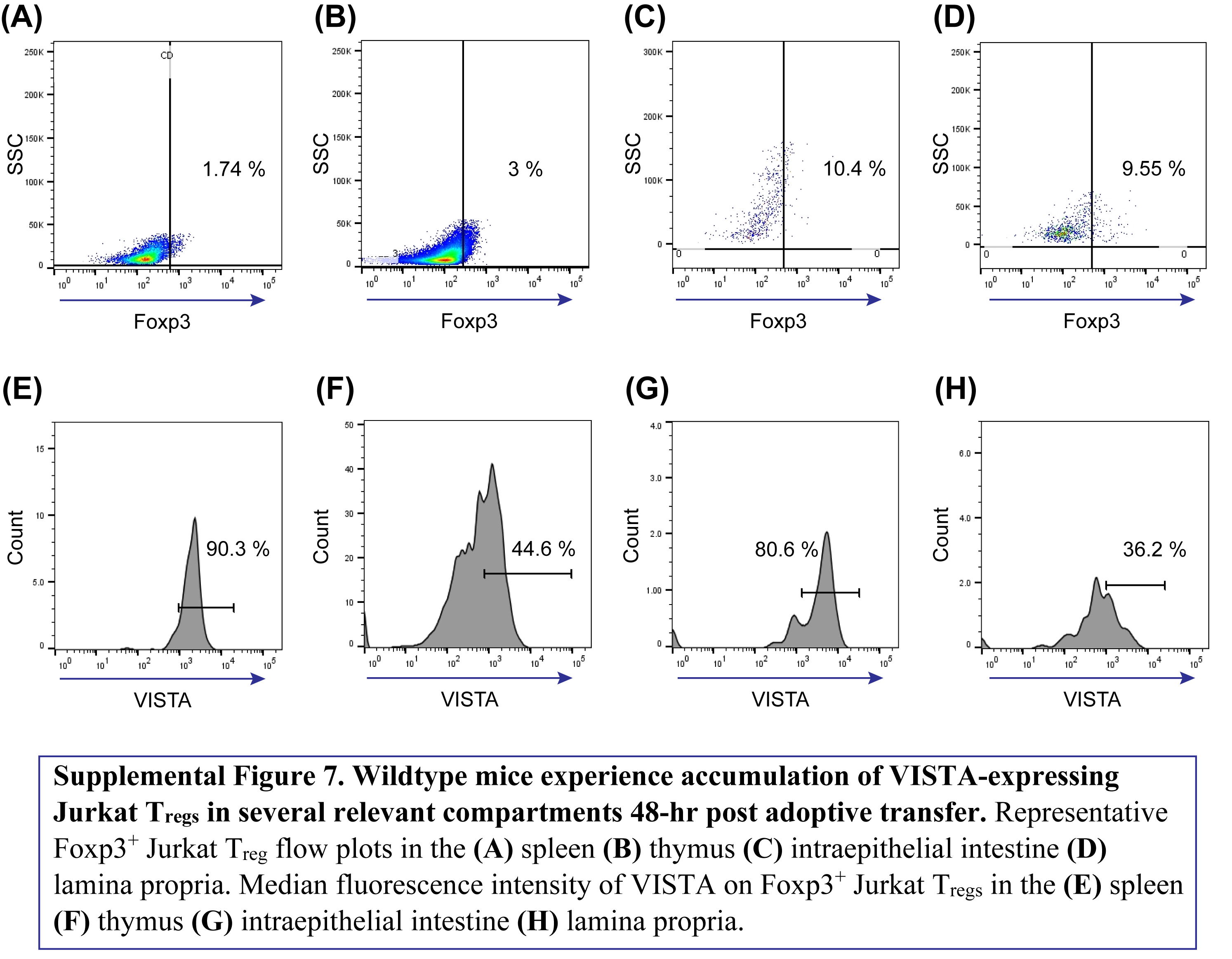

Supplement: Supplementary file 7 [file Image_7.tif]

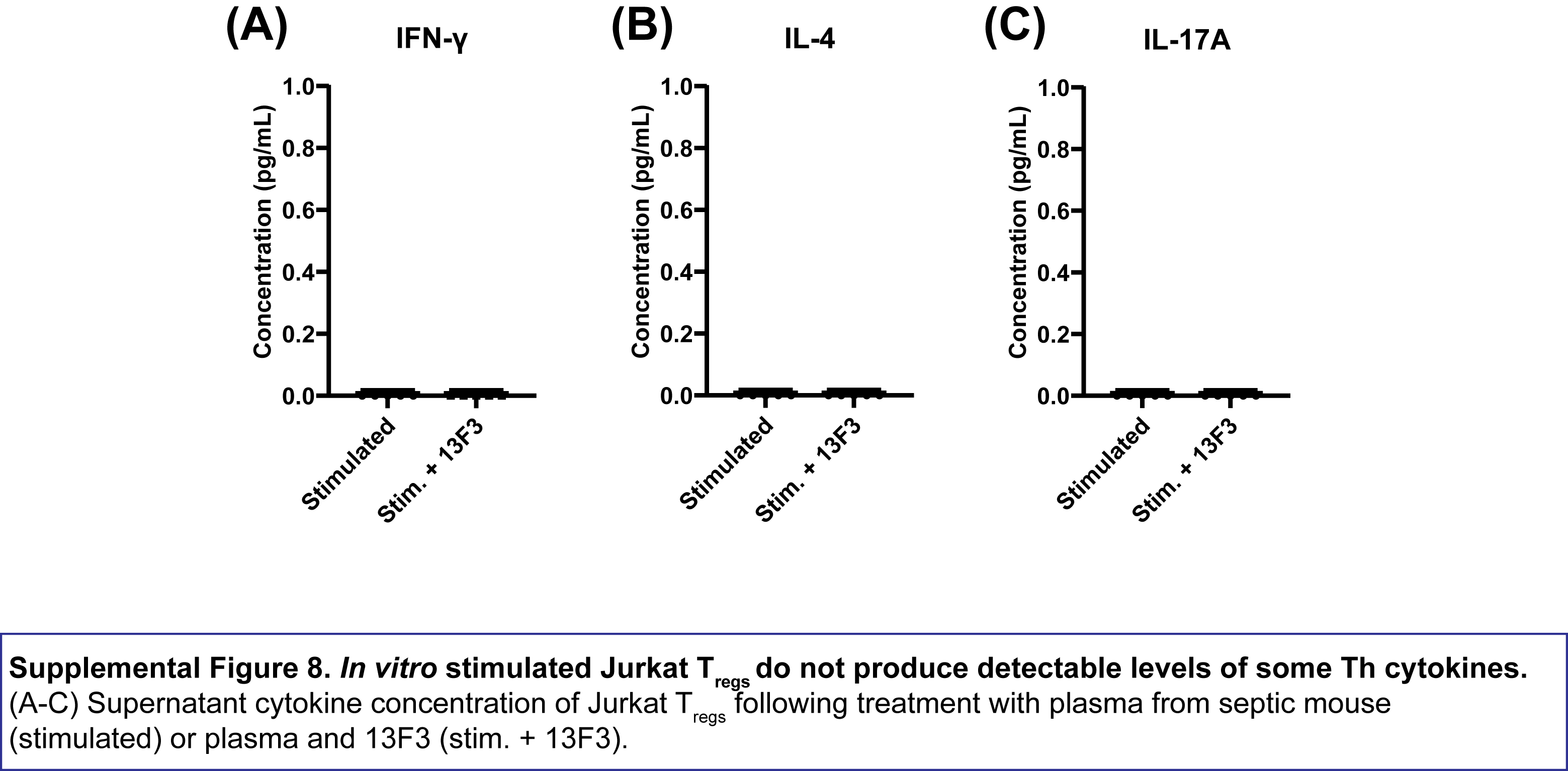

Supplement: Supplementary file 8 [file Image_8.tif]
